# FokI Polymorphism in the Vitamin D Receptor Gene (*VDR)* and Its Association with Lumbar Spine Pathologies in the Italian Population: A Case-Control Study

**DOI:** 10.1371/journal.pone.0097027

**Published:** 2014-05-08

**Authors:** Alessandra Colombini, Marco Brayda-Bruno, Giovanni Lombardi, Samantha Jennifer Croiset, Valentina Vrech, Vincenzo Maione, Giuseppe Banfi, Sabina Cauci

**Affiliations:** 1 Laboratory of Experimental Biochemistry and Molecular Biology, I.R.C.C.S. Istituto Ortopedico Galeazzi, Milano, Italy; 2 Department of Orthopedics and Traumatology – Vertebral surgery III – Scoliosis, I.R.C.C.S. Istituto Ortopedico Galeazzi, Milano, Italy; 3 Department of Medical and Biological Sciences, University of Udine, Udine, Italy; 4 Department of Biomedical Sciences for Health, University of Milano, Milano, Italy; National Cancer Center, Japan

## Abstract

Alterations in vitamin D homeostasis, mainly involving its nuclear receptor (VDR), could have a role in the pathophysiology of the spine. The association between *VDR* polymorphisms and spine disorders has been analyzed in different ethnic groups, focusing on the functional FokI polymorphism. However, so far, inconsistent findings were reported. The aims of this study were to evaluate, in the Italian white population, the *VDR* FokI polymorphism frequencies distribution in subjects with clearly defined lumbar spinal pathologies compared to asymptomatic controls and to analyze the interplay of genetic and conventional risk factors. Using a case-control design, 267 patients with spinal disorders and 220 asymptomatic controls were enrolled, evaluating their exposition to putative risk factors. Patients’ clinical assessment was performed by Magnetic Resonance Imaging. FokI polymorphism (rs2228570) was detected by PCR-RFLP. Genotypes were designated by a lowercase letter (*f* allele, T nucleotide) for the presence of the restriction site and by a capital letter (*F* allele, C nucleotide) for its absence. Family history, higher age and BMI, exposure to vibration, physical job demand, smoking habit and lower practice of leisure physical activity were associated with spinal disorders. The *FF* genotype and *F* allele represented approximately 2-fold risk factors to develop discopathies and/or osteochondrosis concomitant with disc herniation, while *f* allele was protective. In conclusion, the link we observed between *VDR* FokI variants and specific lumbar spine pathologies suggests that spinal tissue degeneration is influenced by the genetic background. Future studies should evaluate the signaling pathways involving alterations in *VDR* and influencing the development and/or progression of spine disorders.

## Introduction

Low back disorders, in particular disc herniation, which represents by far the most prevalent pathology causing pain and sciatica, constitute an important source of disability and one of the most cost-intensive health problems [1]. In Western countries they represent the most common musculoskeletal diseases; it is estimated that 15–20% of adults experience back pain during a single year and around 50–80% have at least one episode during their lifetime [2].

Lumbar disc degeneration (LDD) is considered a primary cause of low back pain (LBP) [3], [4]. Many environmental and behavioral risk factors, including age, gender, weight, occupational load, smoking and exposure to vehicular vibration are likely to contribute to the genesis or to the progression of spinal degeneration and pain onset [5], [6], [7], [8], [9], [10]. In particular, occupational exposures to heavy physical loads, prolonged sitting or non-neutral work postures and vehicle driving have been involved in the disc degeneration processes [11] and considered as the primary source of the mechanical factors damaging the spine [12]. However, some epidemiologic studies and reports among families and twins highlighted that disc herniation, and particularly sciatica, may be explained to a large degree by hereditary factors with apparently a relatively minor effects of environmental and behavioral risk factors [13], [14], [15], [16]. These findings supported the idea that there is a familiar predisposition for development of disc degeneration disorders and that such pathologic conditions may be, at least in part, genetically determined [14], [17], [18], [19], [20], [21], [22], [23].

Vitamin D receptor gene (*VDR*) has been studied as genetic factor putatively predisposing to spine pathologies since 1998 [24], [25]. Several single nucleotide polymorphisms (SNPs) have been identified in the *VDR* sequence, between them FokI (rs10735810, merged into rs2228570) represents an independent polymorphic site [26]. It is a C/T transition polymorphic site located in the *VDR* start codon, affecting the structure and the function of the encoded protein. The allelic variants of this polymorphism code for structurally different receptor proteins from a 424 aminoacids wild-type (*F* allele, C) to a 427 aminoacids long protein (*f* allele, T). The short and long protein forms are associated to a different ability to induce transcription of vitamin D-dependent genes [27], [28], [29]. Consequently, studies concerning the possible association of this SNP with disc degeneration may be particularly interesting for their potential biological significance.

Wide evidences support the notion that the vitamin D endocrine system is involved in the modulation of different biological processes, including skeletal metabolism, immunological response, proliferation and differentiation of a wide variety of cell types [30], [31]. More recently some studies detected the presence of VDR also in the disc cells, highlighting the prominent role of vitamin D active metabolites in the regulation of cells proliferation, matrix genes expression and specific cytokines and proteins production [32], [33].

The pleiotropic effects of vitamin D and its involvement in bony and disc metabolisms could explain why alterations in vitamin D homeostasis could be associated to several pathological conditions of the intervertebral disc (IVD).

The association of FokI polymorphism in *VDR* with hernia, disc degeneration [25], [34], [35], [36] or lumbar spinal stenosis [37] and with occupational vibration, leading to the development of LDD [38], [39], was analyzed in different ethnic groups. However, so far inconsistent findings were reported [40]. This can derive, at least in part, from the lack of a clear definition of the lumbar spine pathological phenotypes and/or by the poor definition or differences associated to the specific ethnic group examined.

To our knowledge, there are no studies investigating the association of FokI polymorphism in *VDR* and specific lumbar spine pathologies in the Italian white population.

Based on these evidences, the aims of this study were:

- to evaluate the *VDR* FokI alleles frequencies distribution in subjects with specific lumbar spine pathologies in comparison with asymptomatic controls in the Italian population;
- to analyze the interplay of genetic and conventional, behavioral and environmental factors in the development of lumbar spine pathologies.

## Materials and Methods

### Ethics Statement

The study was approved by the Institutional Review Board ASL Città di Milano. The methods used in this study were in accordance with the Helsinki Declaration of 1975 as revised in 1996.

### Subjects

Using a case-control design, a total of 487 Italian white subjects, age range 18–65 years, were enrolled after having signed a written informed consent. Inclusion criteria for cases were the presence of a lumbar spine pathology confirmed by Magnetic Resonance Imaging (MRI), while inclusion criteria for controls were the absence of LBP or confirmed severe or chronic spine pathologies. The concomitant presence of other orthopedic diseases such as osteoarthrosis, hip, knee and hand osteoarthritis, osteoporosis was recorded.

Exclusion criteria for both cases and controls were presence of a pathologic condition such as cervical discopathies, scoliosis, fibromyalgia, pregnancy at study enrollment, and chronic diseases like diabetes, cardiovascular diseases, malignancies, lupus erythematosus, and rheumatoid arthritis.

The study included 267 consecutive patients (hospitalized or outpatients) with lumbar spine disorders enrolled for the European Genodisc Project, from May 2009 to January 2013, at the Orthopedics and Traumatology Department of I.R.C.C.S. Istituto Ortopedico Galeazzi (Milan, Italy) by the same clinician. A total of 220 asymptomatic controls were enrolled from January 2011 to January 2013 among healthy volunteers, blood donors or subjects hospitalized for anterior cruciate ligament injuries or hallux valgus surgery.

### Clinical Assessment

**Figure 1:**
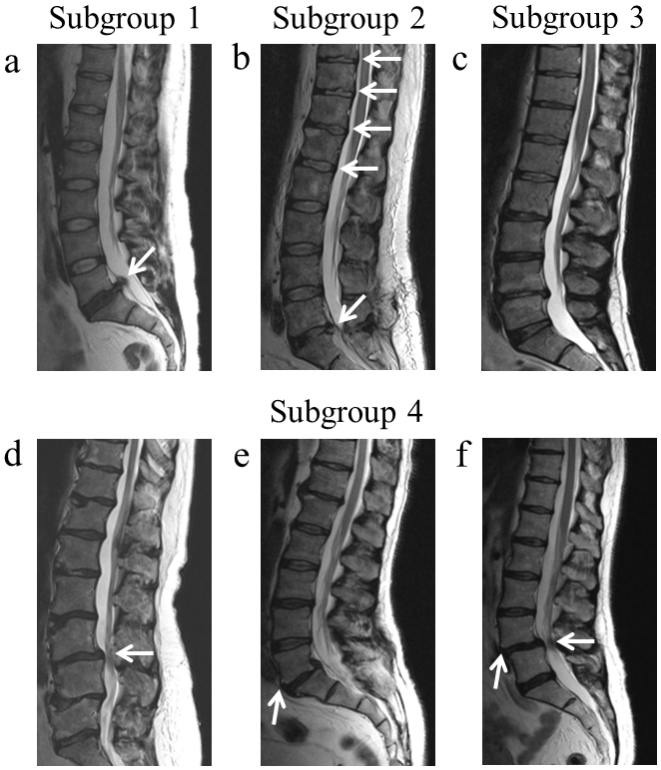
Patient’s clinical assessment and classification in subgroups. a) Subgroup 1, patients with disc herniation only. b) Subgroup 2, patients with discopathies and/or osteochondrosis associated with disc herniation. c) Subgroup 3, patients with discopathies and/or osteochondrosis, without disc herniation, d–f) Subgroup 4, patients with stenosis (d), spondylolisthesis (e) or both (f). White arrows indicate the characteristic pathological features of each subgroup.

Assessment of lumbar spine disorders was performed by an expert clinician in spinal diseases by contrast-enhanced MRI 12 scans of the lumbar spine with a 1.5 T scanner (Avanto, Siemens, Erlangen, Germany). Diagnosis of disc herniation was performed when patients presented disc material protrusion/extrusion beyond the posterior margins of the adjacent vertebral bodies [41] (Figure 1a). Disc herniations were often associated with discopathies and/or osteochondrosis (Figure 1b). Diagnosis of discopathies was performed in presence of degenerative changes of the IVD, while diagnosis of osteochondrosis was performed in presence of degenerative process involving primarily the vertebral bodies structures limiting the disc (disc narrowing, subchondral sclerosis, wavy endplates, osteophytes and Schmorl’s node) [42] (Figure 1c). Spinal stenosis was diagnosed in presence of a narrowing of the central spinal canal, lateral recess or intervertebral foramina [43], [44] (Figure 1d). Finally, patients with degenerative spondylolisthesis presented an acquired anterior displacement of a vertebra over the subjacent one (Figure 1e), due to degenerative changes, without an associated disruption or lysis of the pars interarticularis [45], that is present in patients with lytic/isthmic spondylolisthesis [44]. Stenosis and spondylolisthesis, which are the more concerning structural degenerative spine changes, were often associated (Figure 1f).

Based on these diagnosis, patients were classified in 4 different mutually exclusive subgroups. Subgroup 1 comprised disc herniation only patients (n = 89); Subgroup 2 comprised patients with discopathies (n = 46), osteochondrosis (n = 37) or both (n = 4) associated with disc herniation (total n = 87); Subgroup 3 comprised patients with discopathies (n = 18), osteochondrosis (n = 13) or both (n = 9) without herniation (total n = 40); Subgroup 4 comprised patients with stenosis (n = 11), lytic/isthmic spondylolisthesis (n = 20), degenerative spondylolisthesis (n = 10) or stenosis and lytic/isthmic spondylolisthesis (n = 7), stenosis and degenerative spondylolisthesis (n = 3) (total n = 51).

Since the close linkage between discopathies, disc herniation and osteochondrosis, a further subgroups division from A to D (not mutually exclusive) was performed to better analyze the association between these pathologies and *VDR* FokI genotypes in our cohort of patients: Subgroup A, comprising all herniation cases i. e. Subgroup 1 grouped with Subgroup 2 (total n = 176); Subgroup B, including all discopathies and/or osteochondrosis regardless of herniation, i.e. Subgroup 2 grouped with Subgroup 3 (total n = 127); Subgroup C, comprising all discopathies concomitant with disc herniation (n = 46) grouped with subjects with discopathies alone (n = 18) (total n = 64); and Subgroup D, comprising all osteochondrosis concomitant with disc herniation (n = 37) grouped with subjects with osteochondrosis alone (n = 13) (total n = 50).

### Conventional, Behavioral and Environmental Factors Evaluation

A medical history, including possible low back symptoms or spine surgery, and a questionnaire, reporting the exposition to individual behavioral, environmental, occupational and physical activity putative risk factors were obtained from each subject. The collected information included medical history of family (parents, brothers or sisters), the smoking habit, the job physical demand for the majority of the working years (evaluated by the following score: 0 = sedentary; 1 = light; 2 = medium; 3 = heavy), the hours/day spent driving or as a passenger in motorized vehicles (exposure to vibrations) and, finally, over the past year, the times a week (outside work activity) spent in vigorous physical activity or leisure exercise activities involving twisting, bending or lifting (indicated thereafter collectively as leisure physical activity).

### Determination of Genotypes

Blood samples were collected from the antecubital vein with evacuated ethylenediamine tetra acetic acid (EDTA) tubes (Vacutainer Tubes, Becton-Dickinson, Franklin Lakes, NJ, USA) from the 267 cases and 220 controls. Genomic DNA was extracted from white blood cells according to the procedure of the DNeasy Midi kit (Qiagen, Duesseldorf, Germany). Polymerase chain reaction and restriction fragment length polymorphism (PCR-RFLP) methods were applied to detect the FokI polymorphism of *VDR*. Genomic DNA was amplified using PCR. At first DNA was denatured at 95°C for 5 minutes. Standard PCR conditions were as follows: 94°C for 1 minute, annealing temperature 63°C for 1 minute and 72°C for 2 minutes for 35 cycles and finally 96°C for 1 minute and 72°C for 5 minutes.

The FokI polymorphism of *VDR* was studied using previously tested primers [46].

The resulting 265 bp DNA fragment was digested with FokI restriction enzyme (Euroclone, Milano, Italy) generating two fragments of 196 and 69 bp only in presence of the *f* allele (T). DNA fragments were separated on poliacrylamide gel. Randomly chosen 30 samples’ gel results were confirmed by DNA sequencing.

**Figure 2:**
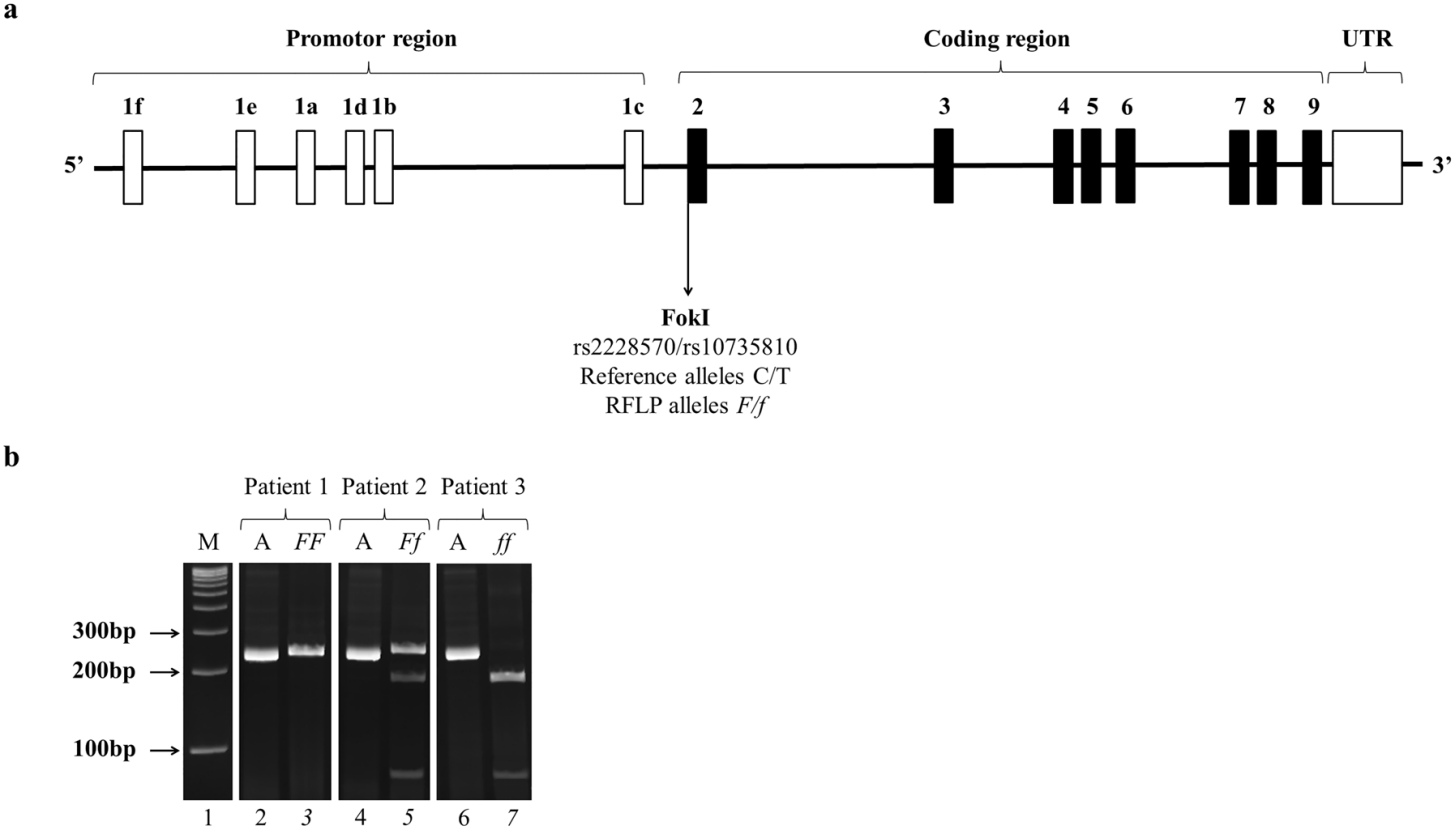
Structure of the genomic region of the *VDR* and location of the FokI polymorphism (a). The *VDR* is located on chromosome 12 and contains nine exons: number 1, designated 1f–1c and indicated with white bars, contains six untranslated subunits in the promotor region, while the other eight exons, numbers 2–9, indicated with black bars in the coding region, encode proteins. FokI polymorphism is located in the exon 2, it is a C>T point mutation, the FokI enzyme cleaves when the restriction site ATG, a start codon, is present. A representative gel for the determination of FokI genotypes in three patients is showed (b). In the first lane there is a molecular weight DNA ladder (M) for size estimation of the DNA fragments. The letter “A” in the second, fourth and sixth lanes indicates the 265 bp PCR amplicons of the three patients. After digestion of the PCR product with FokI restriction enzyme an undigested 265 bp fragment (third lane, homozygous genotype *FF*, CC nucleotides), partially digested 265, 196 and 69 bp fragments (fifth lane, heterozygous genotype *Ff*, CT nucleotides), or totally digested 196 and 69 bp fragments (seventh lane, homozygous genotype *ff*, TT nucleotides) are present for the first, second and third patient, respectively.

Genotypes were designated by a lowercase letter (*f* allele, T nucleotide, mutated) for the presence of the restriction site and by a capital letter (*F* allele, C nucleotide, wild-type) for its absence (Figure 2).

### Statistical Analysis

Kolmogorov-Smirnov test was used to assess the normal data distribution. Student’s t-test or Mann Whitney test were used to assess the differences between the frequency distributions of variables in cases and controls. Odds ratios (ORs) were calculated to set the association between alleles or genotypes and risk of spine pathologies in cases and controls and specific Subgroups of patients. Chi squared or Fisher’s exact test P values were reported as appropriate. Logistic regression was used to evaluate effects of confounders by obtaining adjusted ORs and 95% CIs for genotypes and alleles. Adjusted analysis included conventional risk factors: age, BMI, family history, smoke, physical job demand and exposure to vibrations. Leisure physical activity was not included as confounding because this kind of activity may derive both by personal habits and by absence of pain.

Significance level was held at 0.05. At variance, P values ≤0.10 were considered as a tendency. Statistical softwares used were GraphPad Prism version 5.00 (GraphPad software, La Jolla, CA, USA) and SPSS version 14.0 (SPSS Inc., Chicago, IL, USA).

## Results

### Characteristics of Subjects

The characteristics of the overall population of cases and controls, including age, gender, BMI, spine surgery, family history, smoking habit, job physical demand, exposure to vibrations, practice of leisure physical activity and presence of other orthopedic conditions were shown in Table 1. Among the cases, there were more males (149/267, 55.8%) than females (118/267, 44.2%), while among controls there were almost equal numbers of males (106/220, 48.2%) and females (114/220, 51.8%). Due to the study design, surgery for disc pathologies (39% of cases) and presence of other orthopedic conditions (12.7% of cases) were present only in the cohort of cases.

**Table 1:** Characteristics of the subjects recruited and influence of risk factors of lumbar spine pathologies.

Factors		Controls	All Cases	Subgroup 1	Subgroup 2	Subgroup 3	Subgroup 4
		n = 220	n = 267 (100%)	n = 89 (33.3%)	n = 87 (32.6%)	n = 40 (15.0%)	n = 51 (19.1%)
			P value	P value	P value	P value	P value
Age (Years)	mean ± SD	40.08±9.56	44.19±9.11 P<0.001	42.44±9.27 P = 0.048	43.69±8.90 P = 0.003	42.80±8.33	49.22±8.16 P<0.001
Gender	Males n (%)	106 (48.2)	149 (55.8)	48 (53.9)	55 (63.2) P = 0.018	21 (52.5)	25 (49.0)
	Females n (%)	114 (51.8)	118 (44.2)	41 (46.1)	32 (36.8)	19 (47.5)	26 (51.0)
BMI (kg/m^2^)	mean ± SD	24.26±3.71	25.29±4.06 P = 0.001	24.93±4.05	25.27±3.60 P = 0.005	24.28±3.91	26.76±4.61 P<0.001
Spine surgery	n (%)	/	104 (39.0)	26 (29.2)	29 (33.3)	27 (67.5)	22 (43.1)
Family history	n (%)	35 (15.9)	97 (36.3) P<0.001	33 (37.1) P<0.001	38 (43.7) P<0.001	11 (27.5)	15(29.4) P = 0.028
Smoker	n (%)	92 (41.8)	136 (50.9) P = 0.020	53 (59.5) P = 0.005	50 (57.5) P = 0.014	16 (40.0)	25 (49.0)
Physical job demand^1^ (Score 0–3)	mean ± SD	1.05±0.98	1.38±1.09 P = 0.001	1.41±1.08 P = 0.009	1.38±1.11 P = 0.023	1.40±1.13	1.33±1.05
Exposure to vibrations (Hours/day)	mean±SD	1.38±1.10	2.19±2.56 P = 0.013	2.30±2.90	2.29±2.43 P = 0.001	2.25±2.80	1.78±1.90
Leisure physical activity at least once per week^2^	n (%)	115 (52.3)	85 (32.0) P<0.001	33 (37.5) P = 0.020	25 (28.7) P<0.001	13 (32.5) P = 0.024	14 (27.5) P = 0.002
Other orthopedic conditions^3^	n (%)	/	34 (12.7)	6 (6.7)	13 (14.9)	8 (20.0)	7 (13.7)

1 5 patients had missing information about intensity of physical demand at work, thus a total of 262 data were available. Physical job demand score used: 0 = sedentary; 1 = light; 2 = medium; 3 = heavy.

2 1 patient had missing information about leisure physical activity per week, thus a total of 266 data were available.

3 Orthopedic conditions included: osteoarthrosis, hip, knee and hand osteoarthritis, and osteoporosis.

Subgroup 1 = patients with disc herniation alone; Subgroup 2 = patients with discopathies and/or osteochondrosis associated with disc herniation; Subgroup 3 = patients with discopathies and/or osteochondrosis without herniation; Subgroup 4 = patients with stenosis and/or spondylolisthesis.

Only significant P≤0.05 were indicated.

In our group of patients with lumbar spine disorders the more frequent pathologies were the disc herniation alone (Subgroup 1, 89/267, 33.3%), herniation associated with discopathies and/or osteochondrosis (Subgroup 2, 87/267, 32.6%), followed by stenosis and/or spondylolisthesis without herniation (Subgroup 4, 51/267, 19.1%) and by discopathies and/or osteochondrosis without herniation (Subgroup 3, 40/267, 15.0%). Gender distribution throughout Subgroups 1 to 4 was similar, with the exception of Subgroup 2, which included 55 males (63.2% of male cases) and only 32 females (36.8% of female cases). Family history of spine pathologies was highest (43.7%) in the Subgroup 2. Spine surgery frequency was highest in Subgroup 3 (67.5%).

### Influences of Conventional, Behavioral and Environmental Risk Factors

Associations between lumbar spine pathologies and putative conventional risk factors were reported as continuous variables in all cases and in mutually exclusive Subgroups 1 to 4 in Table 1. In the overall cohort of cases higher age (P<0.001), higher BMI (P = 0.001), family history (OR = 3.02; 95%CI = 1.94–4.68; P<0.001), smoking habit (OR = 1.54; 95%CI = 1.08–2.21; P = 0.020), stronger job physical demand (P = 0.001), higher exposure to vibration (P = 0.013) were all significantly associated with the development of lumbar spine pathologies. Controls subjects practiced leisure physical activity more frequently than the pathological subjects (OR = 2.33; 95%CI = 1.61–3.37; P<0.001).

By analyzing the specific subgroups (from 1 to 4) of cases in comparison with controls, an influence of higher age was evidenced for Subgroup 1 (P = 0.048), Subgroup 2 (P = 0.003) and Subgroup 4 (P<0.001), with a tendency for Subgroup 3 (P = 0.093). An influence of higher BMI was observed only for Subgroup 2 (P = 0.005) and Subgroup 4 (P<0.001). Although not significant, a tendency for an association between male gender and spine pathologies was observed (OR = 1.36; 95%CI = 0.95–1.94; P = 0.094). However, male gender was significantly associated with spine pathologies only in Subgroup 2 (OR = 1.85; 95%CI = 1.11–3.08; P = 0.018).

The association of spine pathologies with family history was observed in Subgroup 1 (OR = 3.11; 95%CI = 1.78–5.46; P<0.001), Subgroup 2 (OR = 4.10; 95%CI = 2.35–7.15; P<0.001) and Subgroup 4 (OR = 2.20; 95%CI = 1.09–4.44; P = 0.028), with a tendency for Subgroup 3 (OR = 2.00; 95%CI = 0.92–4.38; P = 0.081).

Concerning the smoking habit a higher frequency of subjects exposed to this risk factor was registered in pathological Subgroups 1 (OR = 2.05; 95%CI = 1.24–3.38; P = 0.005) and Subgroup 2 (OR = 1.88; 95%CI = 1.14–3.11; P = 0.014) in comparison with controls.

A stronger job physical demand was observed in Subgroup 1 (P = 0.009) and Subgroup 2 (P = 0.023), with a tendency for Subgroup 3 (P = 0.064) and Subgroup 4 (P = 0.091). Finally, a higher exposure to vibrations was registered in Subgroup 2 (P = 0.001) in respect to controls.

Controls subjects practiced leisure physical activity more frequently than the subjects in Subgroup 1 (OR = 1.82; 95%CI = 1.10–3.03; P = 0.020), Subgroup 2 (OR = 2.72; 95%CI = 1.59–4.64; P<0.001), Subgroup 3 (OR = 2.27; 95%CI = 1.12–4.64; P = 0.024) and Subgroup 4 (OR = 2.89; 95%CI = 1.48–5.65; P = 0.002).

### VDR Genotypes and Alleles in Controls and Cases

In our total sample of 487 Italian white subjects the frequency of *FF* homozigotes was 42.3% (206/487), *Ff* heterozigotes was 46.9% (219/487) and *ff* homozigotes was 12.9% (62/487). *F* allele had a frequency of 64.8% (631/974) and the *f* allele had a frequency of 35.2% (343/974). The observed genotype frequencies were consistent with Hardy-Weinberg equilibrium (X^2^ = 0.102, P = 0.75).

Table 2 reported frequencies of FokI genotypes and alleles in cases and controls, including crude and adjusted ORs and 95%CIs. Not significant differences in the frequencies distribution of both genotypes and alleles were observed in overall subjects, the wild homozygous *FF* genotype was present in 43.8% of cases versus 40.5% of controls, while the minor *ff* homozygous genotype was found in 14.5% controls versus 11.2% of cases, and the heterozygous *Ff* genotype had the same frequency between cases and controls (45.0%). *F* allele had a frequency of 66.3% in cases versus 63.0% of controls, while *f* allele had a frequency of 37.0% in controls versus 33.7% of cases. Neither controls (X^2^ = 0.273, P = 0.60) nor cases (X^2^ = 0.0085, P = 0.93) deviated from Hardy-Weinberg equilibrium.

**Table 2:** *VDR* FokI genotypes and alleles in controls and cases.

Variables		Controls n = 220 (%)	Cases n = 267 (%)	Crude OR (95% CI)	Adjusted OR^1^ (95% CI)
*VDR*-FokI genotypes	*FF*	89 (40.5)	117 (43.8)	1.15 (0.80–1.65)	1.10 (0.74–1.63)
	*Ff*	99 (45.0)	120 (45.0)	1.00 (0.70–1.43)	0.98 (0.66–1.45)
	*ff*	32 (14.5)	30 (11.2)	0.74 (0.44–1.27)	0.86 (0.48–1.55)
*VDR*-FokI alleles	*F*	277/440 (63.0)	354/534 (66.3)	1.16 (0.89–1.51)	1.09 (0.82–1.45)
	*f*	163/440 (37.0)	180/534 (33.7)	0.86 (0.66–1.12)	0.92 (0.69–1.23)

1 Adjusted OR: multivariate analysis, OR adjusted for age, BMI, family history, smoke, physical job demand and exposure to vibrations.

### Association of Specific Lumbar Spine Pathologies and VDR Genotypes and Alleles

The frequencies distribution of FokI *VDR* genotypes and alleles in different specific pathological subgroups (1–4 and A–D) and in controls subjects, with the relative crude and adjusted ORs and 95%CIs, were reported in Tables 3 and 4, respectively. Concerning genotypes (Table 3), the only significant association was found between the wild homozygous *FF* genotype and the presence of discopathies and/or osteochondrosis concomitant with disc herniation (Subgroup 2) (crude OR = 1.90; 95%CI = 1.15–3.13; P = 0.012; adjusted OR = 2.09; 95%CI = 1.17–3.72; P = 0.012). In the same patients (Subgroup 2) the *ff* genotype tended to be protective (crude OR = 0.43; 95%CI = 0.17–1.08; P = 0.073), but P became >0.10 after the OR adjustment for conventional risk factors.

**Table 3:** Association of lumbar spine pathologies and *VDR* FokI genotypes.

	*FF*	Crude OR	Adjusted OR^1^	*Ff*	Crude OR	Adjusted OR^1^	*ff*	Crude OR	Adjusted OR^1^
	n (%)	(95% CI)	(95% CI)	n (%)	(95% CI)	(95% CI)	n (%)	(95% CI)	(95% CI)
		P value	P value			P value		P value	P value
Controls n = 220	89 (40.5)			99 (45.0)			32 (14.5)		
Subgroup 1 n = 89	37 (41.6)	1.05 (0.64–1.73)	0.93 (0.53–1.62)	40 (44.9)	1.00 (0.61–1.64)	1.03 (0.60–1.77)	12 (13.5)	0.92 (0.45–1.87)	1.07 (0.49–2.34)
Subgroup 2 n = 87	49 (56.3)	1.90 (1.15–3.13) P = 0.012	2.09 (1.17–3.72) P = 0.012	32 (36.8)	0.71 (0.43–1.18)	0.62 (0.35–1.11)	6 (6.9)	0.43 (0.17–1.08)	0.48 (0.17–1.38)
Subgroup 3 n = 40	14 (35.0)	0.79 (0.39–1.60)	0.72 (0.35–1.57)	23 (57.5)	1.65 (0.84–3.27)	2.00 (0.96–4.16)	3 (7.5)	0.48 (0.14–1.64)	0.30 (0.07–1.24)
Subgroup 4 n = 51	17 (33.3)	0.74 (0.39–1.40)	0.58 (0.28–1.19)	25 (49.0)	1.17 (0.64–2.16)	1.22 (0.62–2.40)	9 (17.6)	1.26 (0.56–2.83)	1.77 (0.71–4.41)
Subgroup 1+2+3 n = 216	100 (46.3)	1.27 (0.87–1.85)	1.22 (0.80–1.85)	95 (44.0)	0.96 (0.66–1.40)	0.97 (0.64–1.46)	21 (9.7)	0.63 (0.35–1.14)	0.68 (0.35–1.30)
Subgroup A n = 176	86 (48.9)	1.41 (0.94–2.10)	1.36 (0.87–2.13)	72 (40.9)	0.85 (0.57–1.26)	0.81 (0.51–1.26)	18 (10.2)	0.67 (0.36–1.24)	0.81 (0.41–1.61)
Subgroup B n = 127	63 (49.6)	1.45 (0.93–2.25)	1.48 (0.91–2.40)	55 (43.3)	0.93 (0.60–1.45)	0.94 (0.58–1.53)	9 (7.1)	0.45 (0.21–0.97) P = 0.042	0.38 (0.15–0.94) P = 0.037
Subgroup C n = 64	37 (57.8)	2.02 (1.15–3.55) P = 0.015	1.85 (1.00–3.42) P = 0.049	22 (34.4)	0.64 (0.36–1.14)	0.66 (0.35–1.23)	5 (7.8)	0.50 (0.19–1.34)	0.55 (0.19–1.64)
Subgroup D n = 50	24 (48.0)	1.36 (0.73–2.52)	1.59 (0.79–3.21)	24 (48.0)	1.13 (0.61–2.09)	1.05 (0.52–2.12)	2 (4.0)	0.24 (0.06–1.06)	0.17 (0.03–0.97) P = 0.046

Subgroup 1 = patients with disc herniation alone; Subgroup 2 = patients with discopathies and/or osteochondrosis associated with disc herniation; Subgroup 3 = patients with discopathies and/or osteochondrosis without herniation; Subgroup 4 = patients with stenosis and/or spondylolisthesis. Subgroup A, Subgroup 1 grouped with Subgroup 2 (i.e. all hernia cases with or without concomitant additional conditions); Subgroup B, Subgroup 2 grouped with Subgroup 3; Subgroup C, all discopathies cases with or without concomitant disc herniation; Subgroup D, all osteochondrosis cases with or without concomitant disc herniation.

1 Adjusted OR: multivariate analysis, OR adjusted for age, BMI, family history, smoke, physical job demand and exposure to vibrations.

Only significant P≤0.05 were indicated.

**Table 4:** Association of lumbar spine pathologies and *VDR* FokI alleles.

	*F*	Crude OR	Adjusted OR^1^	*f*	Crude OR	Adjusted OR^1^
	n (%)	(95% CI)	(95% CI)	n (%)	(95% CI)	(95% CI)
		P value	P value		P value	P value
Controls n = 440	277 (63.0)			163 (37.0)		
Subgroup 1 n = 178	114 (64.0)	1.05 (0.73–1.51)	0.95 (0.64–1.41)	64 (36.0)	0.95 (0.66–1.37)	1.06 (0.71–1.57)
Subgroup 2 n = 174	130 (74.7)	1.74 (1.17–2.57) P = 0.005	1.80 (1.15–2.82) P = 0.011	44 (25.3)	0.57 (0.39–0.85) P = 0.005	0.56 (0.35–0.87) P = 0.011
Subgroup 3 n = 80	51 (63.8)	1.03 (0.63–1.70)	1.07 (0.64–1.81)	29 (36.2)	0.97 (0.59–1.59)	0.93 (0.55–1.57)
Subgroup 4 n = 102	59 (57.8)	0.81 (0.52–1.25)	0.65 (0.40–1.07)	43 (42.2)	1.24 (0.80–1.92)	1.53 (0.94–2.49)
Subgroup 1+2+3 n = 432	295 (68.3)	1.27 (0.96–1.68)	1.22 (0.90–1.66)	137 (31.7)	0.79 (0.60–1.04)	0.82 (0.60–1.11)
Subgroup A n = 352	244 (69.3)	1.33 (0.99–1.79)	1.25 (0.90–1.74)	108 (30.7)	0.75 (0.56–1.01)	0.80 (0.57–1.11)
Subgroup B n = 254	181 (71.3)	1.46 (1.04–2.04) P = 0.026	1.50 (1.04–2.18) P = 0.031	73 (28.7)	0.68 (0.49–0.96) P = 0.026	0.66 (0.46–0.96) P = 0.031
Subgroup C n = 128	96 (75.0)	1.76 (1.13–2.75) P = 0.012	1.62 (1.00–2.62) P = 0.049	32 (25.0)	0.57 (0.36–0.88) P = 0.012	0.62 (0.38–1.00) P = 0.049
Subgroup D n = 100	72 (72.0)	1.51 (0.94–2.44)	1.72 (1.00–2.95) P = 0.051	28 (28.0)	0.66 (0.41–1.06)	0.58 (0.34–1.00) P = 0.051

Subgroup 1 = patients with disc herniation alone; Subgroup 2 = patients with discopathies and/or osteochondrosis associated with disc herniation; Subgroup 3 = patients with discopathies and/or osteochondrosis without herniation; Subgroup 4 = patients with stenosis and/or spondylolisthesis. Subgroup A, Subgroup 1 grouped with Subgroup 2 (i.e. all hernia cases with or without concomitant additional conditions); Subgroup B, Subgroup 2 grouped with Subgroup 3; Subgroup C, all discopathies cases with or without concomitant disc herniation; Subgroup D, all osteochondrosis cases with or without concomitant disc herniation.

1 Adjusted OR: multivariate analysis, OR adjusted for age, BMI, family history, smoke, physical job demand and exposure to vibrations.

Only significant P≤0.05 were indicated.

In subjects with discopathies and/or osteochondrosis without herniation (Subgroup 3) the *Ff* genotype showed a tendency to be a risk factor (adjusted OR = 2.00; 95%CI = 0.96–4.16; P = 0.063) and the *ff* genotype had a tendency to be protective (adjusted OR = 0.30; 95%CI = 0.07–1.24; P = 0.096). No other significant finding was observed even grouping Subgroups 1, 2 and 3 altogether (thus excluding the Subgroup 4).

To further explore possible associations of FokI polymorphisms with specific subsets of patients we grouped in Subgroup A all patients with hernia (with or without concomitant additional conditions), however, no significant genotype differences were evidenced. In Subgroup B we included all patients with discopathies and/or osteochondrosis regardless of hernia presence (Subgroups 2 plus 3), for this subgroup we observed a protective role of the *ff* genotype (crude OR = 0.45; 95%CI = 0.21–0.97; P = 0.042, and adjusted OR = 0.38; 95%CI = 0.15–0.94; P = 0.037). A similar protective association of the *ff* genotype was observed for all patients having osteochondrosis (Subgroup D), (crude OR = 0.24; 95%CI = 0.06–1.06; P = 0.059, and adjusted OR = 0.17; 95%CI = 0.03–0.97; P = 0.046). Finally, by grouping all patients having discopathies (Subgroup C) a 2-fold association was found for the *FF* genotype both in crude and adjusted analyses (crude OR = 2.02; 95%CI = 1.15–3.55; P = 0.015, and adjusted OR = 1.85; 95%CI = 1.00–3.42; P = 0.049).

The alleles distribution (Table 4) showed a higher frequency of the wild *F* allele in Subgroup 2 i.e. patients having discopathies and/or osteochondrosis concomitant with disc herniation (crude OR = 1.74; 95%CI = 1.17–2.57; P = 0.005; adjusted OR = 1.80; 95%CI = 1.15–2.82; P = 0.011). Consequently, the mutated *f* allele was protective for these pathological features. The *F* allele was risky for Subgroup B (discopathies and/or osteochondrosis) crude OR = 1.46; 95%CI = 1.04–2.04; P = 0.026; adjusted OR = 1.50; 95%CI = 1.04–2.18; P = 0.031; and Subgroup C (discopathies in general) crude OR = 1.76; 95%CI = 1.13–2.75; P = 0.012; adjusted OR = 1.62; 95%CI = 1.00–2.62; P = 0.049. Consequently the *f* allele was protective for patients of Subgroups B and C (as reported in Table 4).

## Discussion

To our knowledge, this is the first study that evaluated and showed an association between *VDR* FokI variants and specific spine pathologies in the Italian white population and the largest study showing also the concomitant influences of conventional risk factors.

In our study we determined FokI genotypes and alleles frequencies in a sample of 487 Italian white subjects enrolled in Milan (Northern Italy). Our frequencies were very close to those reported in an other study performed in 102 Italian subjects from Tuscany (Central Italy) (*FF* = 42.2%, *Ff* = 42.2%, *ff* = 15.7%; *F* = 63.2%, *f* = 36.8%) [47]. In general, we observed similar frequencies distribution of FokI genotypes and alleles between our cohort of Italian subjects and European subjects, in particular Finnish twins (n = 85 pairs, *FF* = 28%, *Ff* = 58%, *ff* = 14%; *F* = 60%, *f* = 40%) [25], 56 Finnish controls (*FF* = 44.6%, *Ff* = 46.4%, *ff* = 8.9%; *F* = 67.9%, *f* = 32.1%) [37], 150 Turkish healthy subjects (*F* = 67%, *f* = 33%) [34] and German healthy women (n = 2596, *FF* = 38.5%, *Ff* = 46.3%, *ff* = 15.2%; *F* = 62%, *f* = 38%) [48].

Moreover, we observed that the *VDR* FokI polymorphism in our Italian sample was in Hardy-Weinberg equilibrium as previously found in other European populations [25].

The association between the presence of polymorphisms in the *VDR* and lumbar spine pathologies is a debated topic [24]. In our study, considering the very broad category of patients with lumbar spine disorders, the FokI polymorphism was not associated with disease risk. However, this polymorphism represented a risk factor to develop discopathies in general, and particularly discopathies and/or osteochondrosis concomitant with disc herniation.

Three previous studies from other authors reported an association between the FokI polymorphism in *VDR* and specific signs of disc degeneration in Turkish [34], Brazilian [35] and Finnish [25] populations, with subjects having *Ff* and *ff* genotypes showing a predisposition towards worse phenotypes.

By contrast, other studies found no association between FokI genotypes and disc pathologies. Specifically, no association was observed for disc herniation or lumbar spinal stenosis in the Finnish population [25], [37]; nor for LDD in a Norway case/control study [36] and neither for osteophyte formation without disc degeneration in a cohort of elderly Japanese males and females with LBP [49].

The comparison of data present in the literature is particularly difficult especially because of the study design and ethnic differences in the various research studies. Moreover, the absence of a standardized definition of pathological phenotypes hampers the comparisons and reliable interpretations of the reported data.

In our work, we classified our cases by means of the pathological features evidenced throughout detailed objective evaluation by MRI. This approach allowed us to subgroup patients accordingly to specific lumbar spine pathologies having a defined clinical significance. To evaluate the association of *VDR* FokI genotypes/alleles, we first analyzed the broad sample of all patients with lumbar spine disorders, then we subgrouped them in 4 mutually exclusive subgroups (1 to 4). Moreover, we analyzed subgroups of all patients having a condition in common like hernia, discopathy, osteochondrosis, regardless of other concomitant disorders (Subgroups A to D).

Considering specific subgroup of patients i.e. those suffering from discopathies and/or osteochondrosis concomitant with herniation, and in general all patients having discopathies, the *FF* genotype was associated with a 2-fold increased disease risk, also after adjusting for conventional, behavioral and environmental risk factors. The *F* allele was associated with a 1.5 to 1.8-fold increased risk in all patients having discopathies, in all patients having osteochondrosis, and in patients having discopathies and/or osteochondrosis concomitant with herniation. On the contrary, *f* allele seemed to be protective for these pathological phenotypes. We confirmed these results after adjusting for conventional risk factors.

In general, genetic risk factors may interact with behavioral and environmental factors in enhancing the development of lumbar spine pathologies. In this context, the results present in the literature about a possible interplay between *VDR* FokI genotypes, occupational load exposure and exposition to whole-body vibration were controversial [38], [39].

In our study, regarding environmental risk factors such as exposure to vibrations and job physical effort, we observed an association between lumbar spine pathologies, higher number of hours/day exposure to vibration and higher physical job demand. On the contrary, we noted that the practice of leisure physical activity was inversely associated to lumbar spine pathologies. Due to the study design, we cannot assess if this was a really protective behavior or an indication of absence of major low back pain, concomitant with personal habits. Among the other putative conventional risk factors analyzed, we observed that in our cohort of cases family history, higher age, overweight and smoking habit were associated with risk for lumbar spine pathologies. Overall, our findings highlighted that subject voluntary behaviors in addition to environmental factors are major determinants in lumbar spine pathologies.

A limitation of this study is represented by the difference in the mean age of the recruited cases and controls. We found difficulties in finding over 50 years old healthy subjects and, thus, in the future we would like to enlarge the group of controls, trying to match this difference and to confirm our results in a larger cohort of subjects.

Additionally, an increase in the number subjects with stenosis and/or spondylolisthesis (Subgroup 4), could be useful to perform a better evaluation of particular features of this subgroup.

In conclusion, the conventional, behavioral and environmental factors analyzed in this study represented determinants of risk for the development of lumbar spine pathologies in general. On the contrary, finding of genetic associations required objective characterization of lumbar spine disorders. Particularly, we evidenced that personalized evaluation through imaging techniques of each patient is necessary to determine the appropriate subgroup belonging. Our results showed that patients with *FF* homozygous genotype are at risk to develop discopathies in general and discopathies and osteochondrosis in association with disc herniation, independently by the influence of the conventional behavioral and environmental determinants of risk. It is of note that in our study the *FF* homozigosity was not a risk factor for simple herniation. Notably, the *F* allele was an independent risk factor for all discopathies, discopathies and osteochondrosis, discopathies and osteochondrosis combined with herniation, but not simple herniation, stenosis and spondylolisthesis. Additionally, in an adjusted analysis the *F* allele was a 1.7-fold risk factor for osteochondrosis.

Based on the assumption that the wild *F* allele is producing a more transcriptionally active receptor than the *f* allele, it appears that enhanced vitamin D final effects are favoring discopathies and the severe progression of discopathy and/or osteochondrosis to herniation. Interestingly, a very recent study performed in 140 Iranian subjects with diabetes evidenced that the *VDR ff* genotype may be regarded as “low responders” to vitamin D intake [50].

Thus, it is tempting to speculate that a nutrigenic approach based on specific genotypes may be needed to protect patients with specific lumbar spine disorders.

Finally, the lack of significant finding for the association of simple herniation with the genetic background could reflect the accidental/traumatic origin of this condition and/or the necessity to explore different genetic polymorphisms for this specific disorder.
